# Patterns of hydroxyurea use and clinical outcomes among patients with polycythemia vera in real-world clinical practice: a chart review

**DOI:** 10.1186/s40164-016-0031-8

**Published:** 2016-02-01

**Authors:** Shreekant Parasuraman, Marco DiBonaventura, Kelly Reith, Ahmad Naim, Kristen Concialdi, Nicholas J. Sarlis

**Affiliations:** 1 Incyte Corporation, 1801 Augustine Cut-Off, Wilmington, DE 19803 USA; 2 Kantar Health, 11 Madison Avenue, 12th Floor, New York, NY 10010 USA

**Keywords:** Polycythemia vera, Chart review, Hydroxyurea

## Abstract

**Background:**

Hydroxyurea (HU) is among the most commonly used cytoreductive treatments for polycythemia vera (PV), but previous research and clinical experience suggest that not all patients respond optimally, consistently, or durably to HU treatment. This study investigated patterns of HU use and impact on disease control among patients with PV in real-world clinical practice in the United States.

**Methods:**

Oncologists and hematologists recruited between April and July 2014 reported data from patient charts. Treatment history and disease symptom comparisons between HU subgroups were performed using Chi square tests or one-way analyses of variance for categorical and continuous variables. Other analyses were performed using descriptive statistics.

**Results:**

Overall, 329 physicians participated and provided data on 1309 patients with PV (62.3 % male; mean age = 62.5 years, mean time since diagnosis = 5.2 years). In the 229 (17.5 %) patients who had stopped HU, the most common reasons for HU discontinuation—as assessed by the treating clinician—were inadequate response (29.3 %), intolerance (27.5 %), and disease progression (12.7 %). Among patients currently on HU, a significant proportion had elevated blood cell counts: 34.4 % had hematocrit values ≥45 %, 59.4 % had platelet levels >400 × 10^9^/L, and 58.2 % had WBC counts > 10 × 10^9^/L. Two-thirds (66.3 %) of patients had ≥1 elevated count, 40.3 % had ≥2 elevated counts, and 19.8 % had all 3 counts elevated. The most common PV-related signs and symptoms among all patients were fatigue and splenomegaly.

**Conclusions:**

Although many patients with PV benefit from HU therapy, some continue to have suboptimal control of their disease, as evidenced by persistence of abnormally elevated blood cell counts and the continued experience of disease-related manifestations (signs and symptoms). These data further denote a significant medical need for some patients with PV currently or previously treated with HU.

**Electronic supplementary material:**

The online version of this article (doi:10.1186/s40164-016-0031-8) contains supplementary material, which is available to authorized users.

## Background

Polycythemia vera (PV) is a progressive, chronic myeloproliferative neoplasm characterized by a primary, clonally-driven abnormal increase in red cell mass and elevations in platelet and white blood cell (WBC) counts [1]. The 2008 World Health Organization major diagnostic criteria for PV require erythrocytosis (i.e., elevated hemoglobin, hematocrit, red cell mass levels) or an activating mutation in *JAK2*; minor diagnostic criteria include bone marrow trilineage myeloproliferation, subnormal serum erythropoietin level, and endogenous erythroid colony growth [2]. A confirmed diagnosis per these criteria requires a patient to meet either (1) both major criteria and 1 minor criterion or (2) the first major criterion (i.e., erythrocytosis) and 2 minor criteria. There are over 100,000 patients with PV in the United States [3]. The condition is associated with an elevated risk of thrombosis (both arterial and venous), which is one of the most common causes of death in patients with PV [4]. As the disease progresses, a number of other hematologic complications may evolve, including secondary acute myeloid leukemia, myelodysplastic syndrome, and post-PV myelofibrosis [4, 5]. Although presentation is most common at 60 years of age or older [4, 6], one-third of PV cases occur in patients younger than age 50 [4].

Treatment of PV is aimed at the prevention of thrombotic events [1], and typical frontline management includes a combination of low-dose aspirin and phlebotomy (PBT) to decrease hematocrit (Hct) to <45 % [7, 8]. Management of WBC counts and platelet levels are also important treatment goals [9]. Underscoring the importance of careful management of PV, a study by Marchioli et al. found that treating to a higher Hct target range of 45–50 % versus stringently maintaining a Hct of <45 % was associated with a fourfold increase in death from cardiovascular causes or major thrombosis after a median follow-up of 31 months [7].

Some patients with PV require cytoreductive therapy to achieve target Hct levels [10] and to normalize WBC and platelet counts per European LeukemiaNet (ELN) response criteria [11]. Ruxolitinib is the only agent with regulatory approval for use in the PV setting, indicated by the US Food and Drug Administration for patients with PV who have had an inadequate response to or are intolerant of hydroxyurea (HU; also known as hydroxycarbamide) [12] and by the European Medicines Agency for the treatment of adult patients with PV who are resistant to or intolerant of HU [13]. Interferon-α is used to treat some patients with PV [10] and additional options are currently in development (e.g., other JAK2 inhibitors [14, 15], histone deacetylase inhibitors [16, 17]). Although not approved by regulatory agencies for the treatment of PV, HU is the most commonly used cytoreductive agent in the PV setting [1]. HU can be clinically efficacious; however, some patients may not receive adequate benefit or may not tolerate long-term therapy [18]. Indeed, a study by Alvarez-Larrán et al. found that 12 and 13 % of patients with PV had resistance and intolerance to HU, respectively, with the former significantly associated with an increased risk of death [18]. Thus, about one in four patients with PV who receive treatment with HU fall short of treatment goals. In real-world clinical practice, HU resistance is often secondary (i.e., develops over time). Indeed, the treatment patterns for high-risk patients with PV or those whose disease advances over time can sometimes evolve toward the combined use of HU and PBT. In the latter case, patients whose disease had been adequately controlled by HU end up needing more aggressive management, thus forcing the treating clinician to reintroduce PBT to the ongoing cytoreductive therapy to achieve control of blood cell counts and/or control of disease-related symptom burden [1, 19].

Although several studies have examined PV and its treatment in European populations [7, 18], none to our knowledge have studied the effectiveness and limitations of HU in clinical practice in the United States. Using a retrospective chart review method, this study investigated patterns of HU use and the impact of HU on the signs, symptoms, and complications of PV in a real-world US population.

## Results

### Physicians and patients

A total of 329 physicians participated, with the majority (78.1 %) being hematologist-oncologists (Table 1). These physicians provided information on 1309 patients (Table 2). The majority of patients were men (62.3 %); overall, the mean (SD) age was 62.5 (12.2) years and mean (SD) time from PV diagnosis was 5.2 (2.8) years.

**Table 1:** Demographic characteristics of the survey-participating physicians

	Total physician sample (N = 329)
Sex, n (%)	
Male	256 (77.8)
Female	51 (15.5)
Decline to provide	22 (6.7)
Years of age, n (%)	
<35	11 (3.3)
35‒44	118 (35.9)
45‒54	105 (31.9)
55‒64	70 (21.3)
>65	6 (1.8)
Prefer not to state	19 (5.8)
Office location, n (%)	
Major metropolitan area, population >500,000	116 (35.3)
Urban area, population between 100,000 and 500,000	98 (29.8)
Suburb of a large city, population >100,000	73 (22.2)
Small city, population between 30,000 and 100,000	22 (6.7)
Rural or small town, population <30,000	20 (6.1)
Practice type, n (%)	
Solo practice	23 (7.0)
Single-specialty group practice	189 (57.4)
Multi-specialty group practice	117 (35.6)
Self-reported medical specialty, n (%)	
Hematology	21 (6.4)
Oncology (medical oncologist)	51 (15.5)
Hematology-oncology	257 (78.1)
Years practicing	
Mean ± SD	13.8 ± 6.8
Number of patients with PV under care in past 12 mo	
Mean ± SD	55.1 ± 75.2

*PV* polycythemia vera

**Table 2:** Demographic and health history characteristics of patients in retrieved medical charts

	Total patient sample (N = 1309)
Years of age	
Mean ± SD	62.5 ± 12.2
Sex, n (%)	
Male	816 (62.3)
Female	493 (37.7)
BMI	
Mean ± SD	25.9 ± 4.6
Deceased, n (%)	
No	1291 (98.6)
Yes	18 (1.4)
Race/ethnicity, n (%)	
Non-Hispanic white	864 (66.0)
Non-Hispanic black	171 (13.1)
Hispanic	161 (12.3)
Multiracial/other	82 (6.3)
Unknown race/ethnicity	31 (2.4)
Employment status, n (%)	
Employed	496 (37.9)
Retired/unemployed	629 (48.1)
On disability	59 (4.5)
Unknown	125 (9.6)
Insurance status, n (%)	
Private insurance	562 (42.9)
Medicare	500 (38.2)
Medicaid	126 (9.6)
VA	36 (2.8)
State health exchange	33 (2.5)
Uninsured	27 (2.1)
TRICARE	16 (1.2)
Unknown	106 (8.1)
Charlson comorbidity index	
Mean ± SD	0.6 ± 0.8
Number of treated comorbidities	
Mean ± SD	1.6 ± 1.2

*BMI* body mass index, *VA* veterans affairs

### Hydroxyurea treatment patterns

**Fig. 1:**
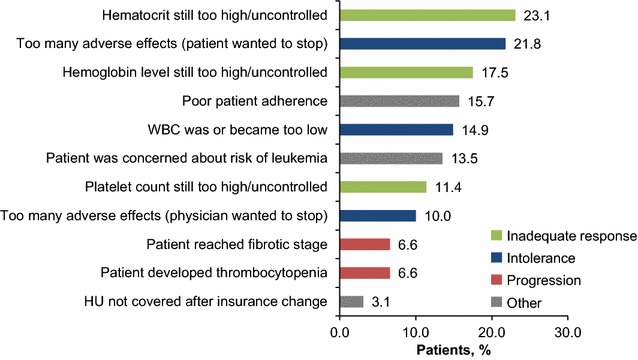
Reasons for HU discontinuation (n = 229). *HU* hydroxyurea, *WBC* white blood cell. Please note that these reasons were not mutually exclusive, and the sum of the percentages is greater than 100 %

A total of 1080 (82.5 %) patients were still using HU at the time of chart abstraction, whereas 229 (17.5 %) had discontinued HU. Reasons for discontinuation were related to inadequate response (abnormally elevated Hct, hemoglobin, or platelet counts; 29.3 %), HU intolerance (i.e., adverse effects; 27.5 %), or disease progression (fibrotic stage PV, persistent thrombocytopenia; 12.7 %); each individual patient could be assigned ≥1 reason for discontinuation (Fig. 1).

As shown in Table 3, the mean daily dose of HU was similar in patients currently and previously treated with HU (approximately 1 g); the highest, lowest, and most stable HU doses were also similar between groups. Mean (SD) duration of HU use was 47.0 (30.8) months among patients currently using HU (31.4 % had been on HU for at least 1 year) and 23.2 (24.5) months among those who had discontinued HU. Among patients who discontinued HU, the mean (SD) time since HU discontinuation was 20.2 (26.4) months.

**Table 3:** HU-related treatment patterns associated with patients who discontinued and those who did not

	Currently using HU therapy (n = 1080)	Discontinued HU therapy (n = 229)
Years diagnosed with PV		
n	1080	229
Mean ± SD	5.23 ± 2.77	5.25 ± 2.82
Duration of HU therapy for current users, mo		
n	1080	–
Mean ± SD	46.97 ± 30.83	–
Duration of HU therapy before discontinuation, mo		
n	–	229
Mean ± SD	–	23.19 ± 24.45
Current HU dose, mg/d		
n	1055	–
Mean ± SD	984.2 ± 673.6	–
Median (q25–q75)	1000.0 (500.0–1000.0)	–
Last dose of HU before discontinuation, mg/d		
n	–	225
Mean ± SD	–	990.9 ± 689.3
Median (q25–q75)	–	1000.0 (500.0–1200.0)
Highest ever HU dose, mg/d		
n	491	123
Mean ± SD	1413.0 ± 875.5	1443.6 ± 876.4
Median (q25–q75)	1000.0 (1000.0–2000.0)	1000.0 (1000.0–2000.0)
Lowest ever HU dose, mg/d		
n	503	125
Mean ± SD	745.1 ± 576.8	744.0 ± 437.9
Median (q25–q75)	500.0 (500.0–1000.0)	500.0 (500.0–1000.0)
Most stable HU dose, mg/d		
n	468	114
Mean ± SD	1129.8 ± 742.2	1167.2 ± 781.7
Median (q25–q75)	1000.0 (712.5–1500.0)	1000.0 (500.0–1500.0)
Number of HU dose adjustments in 3 mo before discontinuation		
n	–	205
Mean ± SD	–	0.48 ± 1.06

*HU* hydroxyurea, *PV* polycythemia vera

### Clinicohematologic response while on hydroxyurea

**Fig. 2:**
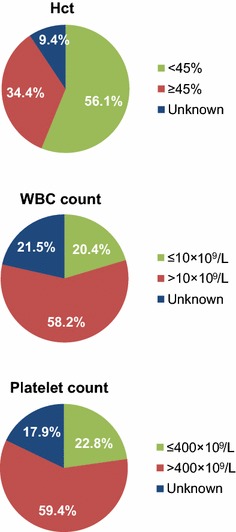
Most recent laboratory values among patients currently using HU (n = 1080). *Hct* hematocrit, *HU* hydroxyurea, *WBC* white blood cell. Hct ≥45 %, WBC counts >10 × 10^9^/L, and platelet counts >400 × 10^9^/L, were all considered elevated values for patients with PV

**Fig. 3:**
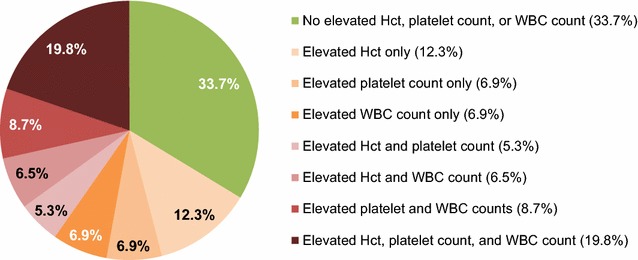
Percentage of patients currently using HU (n = 1080) with different combinations of elevated laboratory values. *Hct* hematocrit, *HU* hydroxyurea, *WBC* white blood cell. Hct ≥45 %, WBC counts >10 × 10^9^/L, and platelet counts >400 × 10^9^/L, were all considered elevated values for patients with PV

**Fig. 4:**
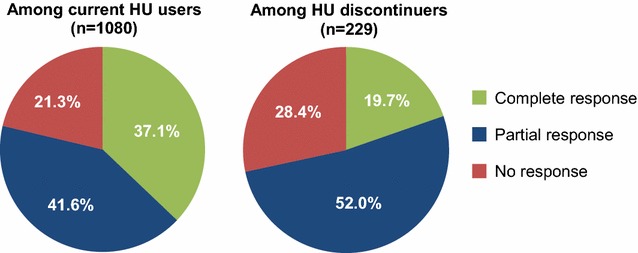
Distribution of CHR status among patients currently receiving HU and patients who discontinued HU. *ELN* European LeukemiaNet, *HU* hydroxyurea. CHR status was determined per ELN response criteria. ELN response criteria are based on the following: levels of Hct, platelet count, WBC count, spleen size, and disease symptoms (pruritus, angina, headache) [9]

**Fig. 5:**
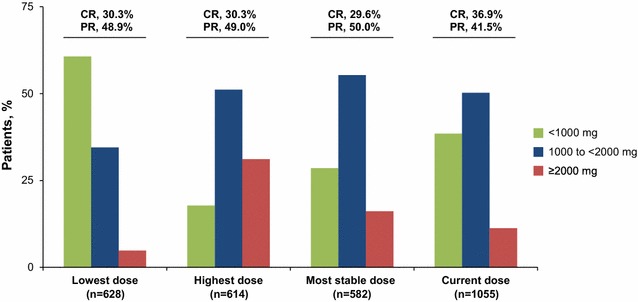
Distributions of the lowest, highest, most stable, and current HU doses with corresponding response rates. *CR* complete response, *PR* partial response

Although patients currently on HU had been treated with HU for a mean (SD) of 47.0 (30.8) months, a notable proportion had elevated blood cell counts above response thresholds: 34.4 % had Hct levels ≥45 % , 58.2 % had WBC counts >10 × 10^9^/L, and 59.4 % had platelet counts >400 × 10^9^/L (Fig. 2). Two-thirds (66.3 %) of these patients had ≥1 elevated value, 40.3 % had ≥2 elevated values, and 19.8 % had all 3 values elevated (Fig. 3). Only 37.1 % of patients currently on HU had achieved a complete response (CR) per European LeukemiaNet (ELN) criteria; 41.6 % had achieved a partial response (PR), whereas 21.3 % had no response; this was significantly different than patients who had discontinued HU (19.7, 52.0, 28.4 % of whom had a CR, PR, and no response, respectively; *P* < 0.05) (Fig. 4). Furthermore, a relationship did not appear to exist between HU dose and CR or PR status (Fig. 5), although the disease severity may have been worse among patients receiving more intense HU dose regimens.

### Polycythemia vera‒related signs and symptoms

The most commonly observed PV-related signs and symptoms ever experienced among all patients were fatigue (62.8 %) and splenomegaly (58.3 %). The prevalence of PV-related signs, symptoms, and events in the past 12 months were summarized in 2 HU therapy-defined subgroups: (1) since starting HU therapy among patients currently using HU and (2) in the past 12 months among patients who discontinued HU. Several symptoms were significantly more common among patients still using HU therapy versus those who had discontinued HU (*P* < 0.05; Table 4), including dizziness (10.4 vs 17.9 %), abdominal discomfort (9.8 vs 17.0 %), and bone pain (4.0 vs 7.0 %).

**Table 4:** Incidence of signs, symptoms, and events in the past 12 months

Sign, symptom, or event, n (%)	Currently using HU therapy (n = 1080)	Discontinued HU therapy (n = 229)
Fatigue or tiredness	304 (28.1)	74 (32.3)
Splenomegaly	267 (24.7)	60 (26.2)
Plethora	138 (12.8)	27 (11.8)
*Dizziness*	*112* (*10.4*)	*41* (*17.9*)*
Headache	108 (10.0)	23 (10.0)
*Abdominal discomfort/pain*	*106* (*9.8*)	*39* (*17.0*)*
Hepatomegaly	103 (9.5)	27 (11.8)
Pruritus	96 (8.9)	23 (10.0)
Feeling of fullness	89 (8.2)	28 (12.2)
Thrombotic events^a^	64 (5.9)	11 (4.8)
Cardiovascular problems	52 (4.8)	14 (6.1)
*Bone pain*	*43* (*4.0*)	*16* (*7.0*)*
*Anemia*	*28* (*2.6*)	*13* (*5.7*)*
Thrombophlebitis	24 (2.2)	3 (1.3)
Thrombosis	22 (2.0)	2 (0.9)
Neutropenia	21 (1.9)	3 (1.3)
Thrombocytopenia	14 (1.3)	4 (1.8)
Stroke/TIA	18 (1.7)	5 (2.2)
Pulmonary embolism	7 (0.6)	1 (0.4)
Cancer	5 (0.5)	0
Elevation in blasts	2 (2.1)	4 (1.8)
*Unknown*	*21* (*1.9*)	*12* (*5.2*)*
No new symptoms	471 (43.6)	91 (39.7)

*HU* hydroxyurea, *TIA* transient ischemic attack

Values in italics identify signs, symptoms, or events that were significantly (*P* < 0.05) different between patients currently using HU therapy and those who discontinued HU therapy

* *P* < *0.05*

^a^Includes all patients who experienced pulmonary embolism, stroke/TIA, thrombophlebitis, and/or thrombosis; patients may have experienced more than 1 thrombotic event

## Discussion

Patients with PV experience a significant disease burden that can interfere with their quality of life and ability to engage in daily activities [20, 21]. Patients are also at significant increased risk for morbidity and mortality, including the risk for thromboembolic events [4, 22]. Despite the established importance of maintaining Hct levels <45 % using traditional treatment approaches such as HU cytoreduction to avoid these adverse outcomes, more than one-fourth of patients do not achieve this Hct target [7, 8]. The current report examines real-world treatment patterns among a large, representative sample of patients with PV currently or previously treated with HU across multiple treatment centers in the US.

Nearly one-fifth of patients in this study discontinued HU therapy, mostly because of a lack of response or safety/tolerability. Using our definition of response (based on applying published ELN criteria to the data collected in this study [9]), approximately 80 % of patients achieved either a CR or PR while on HU treatment. These results suggest that while many patients derive benefit with HU, there is a substantial subset of patients (including patients on HU who had no response and patients who discontinued HU), whose disease remains suboptimally controlled with HU in every-day clinical practice. This is important because the need for HU therapy itself arises when PBT alone becomes inadequate to manage blood cell counts. Indeed, in our cohort, in which all patients were treated with HU for ≥2 months, a large proportion of patients experienced Hct levels above the recommended threshold of 45 %. It is highly likely that such patients required combined therapy with PBT and continued HU. Moreover, the majority of HU users had elevated WBC or platelet counts; the latter, of course, are typically not influenced by the addition of PBT to HU treatment, which further exemplifies the pragmatic management challenges associated with this setting. Evidence suggests that patients with elevated Hct or elevated WBC count are at a significantly higher risk for cardiovascular morbidity and mortality [4, 7]. Patients treated to a higher Hct target range of 45–50 % had 4 times the rate of death from cardiovascular causes or major thrombosis versus patients who were strictly treated to a Hct target of <45 % [7]. Previously, Tefferi et al. identified a significant association between WBC count and subsequent survival [4]. More recently, analysis of data from the prospective Cytoreductive Therapy in Polycythemia Vera (CYTO-PV) trial demonstrated that risk of major thrombosis was approximately four times greater in patients with WBC counts ≥11 × 10^9^/L versus <7×10^9^/L (*P* = 0.02) [23], further highlighting the importance of proper disease control in patients with PV.

In this study, clinicohematologic response (CHR) rates did not correlate with HU dose, suggesting that at least some patients may be intrinsically resistant to HU. Further, more than 30 % of patients had received ≥2000 mg per day of HU as their highest dose, and more than 10 % of patients were being administered ≥2000 mg per day as their current dose, suggesting that even at these very high doses of HU patients do not achieve their treatment goals.

More than half of patients with PV experienced PV-related signs and symptoms after diagnosis, particularly fatigue and splenomegaly. Although the time frame and the method of assessment (physician-reported data from available chart information vs prospective validated patient-reported symptom assessments) were different in our study compared with past studies, the most frequent symptoms were similar (e.g., fatigue, splenomegaly, pruritus, headache, and abdominal pain) [21, 24, 25]. Additionally, Vannucchi et al. reported that the use of standard therapy (HU for the majority of patients) had little effect on reducing symptom scores in patients with PV; in fact, several symptom scores actually increased over a 32-week period in patients with PV treated with traditional therapies [24]. These findings reinforce the important unmet need of symptom control among many patients with PV.

Our study has various limitations consistent with the nature of a retrospective chart review. Patient charts were occasionally incomplete, leading to missing documentation of possible responses (e.g., unknown laboratory values) and potential post hoc interpretation by physicians (e.g., the presence of fatigue), which could have introduced error. In addition, HU dosing was captured at designated time frames, which may not reflect the complete, detailed evolution of a dosing regimen over time for a given patient. Also, although the sample source was demographically representative of the hematologist-oncologist population based on American Medical Association statistics, the patient charts selected may have differed from the overall population of patients across the US with respect to disease severity or the nature, duration, or intensity of applied treatments. Despite these limitations, this analysis provides a contemporary real-world assessment of the patterns of HU use and the corresponding impact on disease control in a large population of patients with PV from multiple academic and community practices across the US.

## Conclusions

Consistent with other reports, this study demonstrates that HU has an important place and role in the treatment of PV in the US. However, a significant subset of patients continue to experience suboptimal control of their PV, as evidenced by persistent elevations in their blood cell counts and the continued presence of burdensome and unremitting disease-related symptoms. Based on our results, it is estimated that for up to a third of patients, PV may be or become suboptimally controlled by HU over the next 5 years. At the time of this study, physicians had few treatment alternatives to consider for PV that was inadequately controlled with HU, even with the concomitant addition of PBT. Therefore, these data further illustrate a significant medical need for a sizeable proportion of patients with PV either currently or previously treated with HU.

## Methods

### Survey development

Initial qualitative interviews were conducted with 19 oncologists, hematologists, and hematologist-oncologists to help inform the development of the chart review survey. A specific focus of these interviews was to understand how clinicians manage patients with PV and clinicians’ experiences with HU therapy. These consisted of 1-h individual in-person detailed interviews conducted by a trained moderator using a structured discussion guide.

### Physician respondents

Physicians were recruited for this study between April and July 2014 to complete an online collation of data gathered from a retrospective chart review. Recruitment was conducted via email from a nationally-representative online panel of board-certified hematologists, oncologists, and hematologist-oncologists who had agreed to participate in blinded periodic research surveys. The recruitment of panel members was not solely based on convenience, as an attempt was made to match the characteristics of the broader population of the American Medical Association specialized in the treatment of cancer or blood disorders (see Additional file 1: Table S1). Physicians were eligible if they spent ≥50 % of their time on direct patient care and had ≥5 patients with PV under their care in the past 12 months, at least 25 % of whom had current or prior HU exposure. Physicians were compensated for their participation.

### Patient charts

Participating physicians selected 4 charts for patients meeting the following criteria: age ≥18 years, alive at or deceased within the past 6 months from the time of chart abstraction, previously diagnosed with PV per physician judgement, with a disease duration of 3–15 years, having received HU therapy for ≥2 months within the last 5 years, having medical record data 12 months both before and after HU initiation, and having not been part of a PV-related clinical trial.

### Chart review survey

Physicians entered all of their responses into an online data collection tool. This retrospective chart review first consisted of questions—completed by each physician in approximately 5 min—on general features of their clinical management of PV, along with demographic (e.g., age, sex) and practice information (e.g., specialty, years in practice, population density of catchment area, etc.).

Physicians then addressed questions regarding each patient. This information included patient demographics (e.g., age, sex, race/ethnicity, employment status, and insurance) and general health history information, such as body mass index and comorbidities.

PV-specific questions included the number of years since initial diagnosis and the various signs/symptoms (e.g., splenomegaly, fatigue), clinical events (e.g., stroke), qualitative laboratory anomalies (e.g., anemia, blast elevation), and discrete/measured laboratory test parameters (i.e., Hct, WBC counts, and platelet counts) that were present at 3 time-points for each patient: initial diagnosis, following the initiation of HU treatment, and during the past 12 months.

Physicians also answered several questions regarding HU treatment, including whether patients were still on HU or had discontinued HU (and how long ago), along with the highest, lowest, and most stable (the latter left up to the interpretation of the physician) doses of HU, if this information was available/retrievable. For patients still using HU, their current dose was reported, whereas for patients who had discontinued HU, their dose just prior to discontinuation was collected.

### Clinicohematologic response

A CHR variable was determined for each patient. The CHR variable was modeled after the ELN definition [9] but was limited by the nature of the data collected in this study (e.g., absence of documentation of the duration of spleen/symptom resolution, absence of or poor documentation of the presence of hemorrhagic or thrombotic events over the follow-up period, and no availability of bone marrow serial specimens necessary to document histologic remission). Specifically, a CR in this analysis was defined as the combination of all the following target criteria being met: Hct <45 %, platelet count ≤400 × 10^9^/L, WBC count ≤10 × 10^9^/L, achievement of a normal spleen size (i.e., lack of an enlarged spleen as reported by the physician), and absence of disease-related symptoms (i.e., pruritus, fatigue, or headache). Patients who did not meet all of the criteria for a CR but had Hct <45 % without concomitant PBT or had a response in 3 or 4 of the 5 CR criteria previously mentioned were considered to have a PR. All others were considered to have no response.

### Statistical analysis

Physician and patient demographics and health history were reported descriptively using means and standard deviations for continuous variables and frequencies and percentages for categorical variables. Treatment history and disease symptoms were compared between those who discontinued and those who were currently treated with HU using Chi square tests and one-way analyses of variance for categorical and continuous variables, respectively. CHR and the achievement of individual blood count target thresholds were reported descriptively (frequencies and percentages).

### Ethics, consent, and permissions

The study was conducted in compliance with the Declaration of Helsinki. All study materials were reviewed and approved by an independent institutional review board (Sterling IRB [Atlanta, GA, USA]; protocol number, 161101176).

## Additional file

**Additional file 1: Table S1.** Demographic Differences Between the Online Panel Source for This Study (“Study Panel”) and the AMA.

## Abbreviations

CHR: clinicohematologic response
CR: complete response
ELN: European LeukemiaNet
Hct: hematocrit
HU: hydroxyurea
PBT: phlebotomy
PR: partial response
PV: polycythemia vera
WBC: white blood cell
