# Supplementary material for: Patterns of hydroxyurea use and clinical outcomes among patients with polycythemia vera in real-world clinical practice: a chart review
Source: Exp Hematol Oncol. 2016 Feb 1;5:3. doi: 10.1186/s40164-016-0031-8 (PMC4736254; doi:10.1186/s40164-016-0031-8)
Supplement: Supplementary file 1 — Additional file 1: Table S1. Demographic Differences Between the Online Panel Source for This Study (“Study Panel”) and the AMA. [file 40164_2016_31_MOESM1_ESM.docx]

**Patterns of Hydroxyurea Use and Clinical Outcomes Among Patients With Polycythemia Vera in Real-World Clinical Practice**

Shreekant Parasuraman, PhD,^1^ Marco DiBonaventura, PhD,^2^ Kelly Reith, MS, MBA,^1^ Ahmad Naim, MD,^1^ Kristen Concialdi, BA,^2^ Nicholas J. Sarlis, MD, PhD^1^

**Supplementary Appendix**

**Table S1. Demographic Differences Between the Online Panel Source for This Study (“Study Panel”) and the AMA**

|  | **Study Panel** | **AMA** |
| --- | --- | --- |
| Region, % |  |  |
| Midwest | 19 | 18 |
| Northeast | 30 | 26 |
| South | 31 | 35 |
| West | 20 | 21 |
| Sex, % |  |  |
| Male | 83 | 79 |
| Female | 17 | 21 |
| Years of age, % |  |  |
| <40 | 6 | 6 |
| 41‒50 | 21 | 13 |
| 51‒60 | 29 | 30 |
| 61‒70 | 36 | 35 |
| >70 | 7 | 16 |
| Years in practice, % |  |  |
| <5 | 1 | 2 |
| 6‒15 | 17 | 11 |
| 16‒25 | 24 | 22 |
| 25‒30 | 15 | 16 |
| >30 | 43 | 49 |

AMA=American Medical Association.
